# Computed tomography for four‐dimensional dose calculation: Effect of detector array length

**DOI:** 10.1002/acm2.70550

**Published:** 2026-03-31

**Authors:** Inhwan Yeo, Qianyi Xu

**Affiliations:** ^1^ Department of Advanced Radiation and Proton Therapy Fairfax Virginia USA; ^2^ Radiation Oncology Thomas Jefferson University Philadelphia Pennsylvania USA

**Keywords:** 16 cm‐length detector for 4DCT, 4D dose, respiratory artifacts

## Abstract

**Purpose:**

Four‐dimensional computed tomography (4DCT) is susceptible to a geometrical error when respiration changes upon patient shift. 4DCT using 16‐cm detector arrays, not needing the shift, has been shown to improve geometrical accuracy, compared with a conventional 4DCT (4‐cm array), albeit with some Hounsfield unit discrepancies. The 4DCTs were validated for 4D planning.

**Materials and methods:**

A lung‐mimicking phantom containing a spherical target with ten respiratory traces and three tumor‐shaped targets with their traces was respectively imaged when, to each of the ten‐positions/phases, the lung was moved (1) step‐wisely by helical scan at each movement (ground truth; 4DCT_GT_), (2) continuously by 4D scan with 16 cm detectors (4DCT_16_), and (3) similarly with 4 cm detectors (4DCT_4_). Respiratory irregularities affected 4DCT_4_ only due to shifts. Treatment plans included volumetric modulated arc therapy (VMAT) and intensity‐modulated proton therapy (IMPT) on averaged CT images from 4DCT_16_ and 4DCT_4_. IMPT was targeted to internal gross target volumes (iGTVs) with density overrides (1.0 g/cm^3^), while VMAT to iGTVs+3 mm, with the objective *V*_prescribed dose(PD)_ ≥ 95%. The plans were recalculated on each phase image of 4DCT_GT_ and those of 4DCT_16_ were additionally calculated on its phase images. The calculated doses were registered to the maximum‐exhale image, and summed.

**Results:**

(1) 4DCT_16_: IMPT plans met the objective, despite under‐coverages in certain phase images, achieving 98.95 ± 1.09% and 98.27% in averaged *V*_PD_ for the spherical and tumor targets, respectively. VMAT plans showed 97.26 ± 1.60% and 94.41% for the respective targets. (2) 4DCT_4_: IMPT plans did not meet the objective with 93.83 ± 9.29% and 97.38% for them. VMAT plans yielded 93.20 ± 5.81% and 91.17% for them. (3) 4DCT_16_ versus 4DCT_GT_: 4D doses based on 4DCT_16_ matched those on 4DCT_GT_ with 62.76 Gy versus 62.76 ± 0.19 Gy for IMPT plans and 63.79 Gy versus 63.90 ± 0.28 Gy for VMAT plans.

**Conclusions:**

The 4DCT_16_ demonstrated accurate and reliable imaging of a lung tumor in 4D dose by IMPT plans. However, it could be affected by phase‐sorting uncertainty.

## 1 INTRODUCTION

Four‐dimensional (4D) dose calculation in a moving tumor is crucial for assessing the deliverable treatment dose in treatment planning.^1^, ^2^, ^3^, ^4^, ^5^, ^6^, ^7^ This calculation utilizes phase images of 4D computed tomography (CT). A conventional 4DCT scanner equipped with short‐length detectors, such as 4 cm requires patient shifts to cover the entire disease site. This scanner is referred to as 4DCT_4_ to distinguish it from 4D dose, although referred to as 4D_4_ in our preceding work.^8^ However, the scanning cannot be done properly and accurately due to its geometrical limitation. The limitation causes structure misses or image artifacts near the junction of patient shift when respiratory pattern changes before and after the repositioning.^8^, ^9^, ^10^, ^11^, ^12^, ^13^, ^14^, ^15^, ^16^, ^17^, ^18^ Important anatomical structures such as targets or diaphragm may be inaccurately captured or distorted. Furthermore, the shifts may also be limited by tube heating.

On the contrary, a 4DCT with long detectors, such as 16 cm (4DCT_16_), may eliminate the need for patient shifting (reduce it, if the desired scan length is greater than 16 cm), and therefore, the errors. The study of 4DCT based on such long‐detectors remains limited.^8^, ^19^ Our preceding study found that 4DCT_16_ provides geometrically accurate images comparable to that of ground‐truth (GT) images in each phase image without significant geometrical misses observed in 4DCT_4_.^8^ However, it also provided images with deviations in Hounsfield units (HUs) exceeding 20 in targets of certain phases from those in GT images. The GT images were provided with nominal helical CT scans of a moving phantom, held static at each position of a respiratory phase, thereby not involving the respiration‐related artifacts, motion averaging, and phase sorting errors. Named as 4DCT_GT_, the images therefore represented tumor volumes in each phase realistically and accurately, serving as the most reliable reference for them. Note that reportedly, a difference in HU of 20 can generate a dose difference of 1%.^20^, ^21^

Provided with these results (geometry and HU), we aim to investigate the impact of them on the 4D dose, and validate 4DCT_16_ dosimetrically for 4D dose planning calculations. To achieve this, the plans that are based on integrated (or internal) gross tumor volumes (iGTVs), derived from 4DCT_16_, were recalculated in the phase‐specific GT images. The resulting phase‐specific doses were summed to generate the 4D deliverable dose. The process was repeated using iGTV from 4DCT_4_ for comparison. In addition, the accuracy of the phase images of 4DCT_16_ was investigated by using them instead of the GT images for the recalculations and comparing the resulting 4D dose with that based on 4DCT_GT_. Note that not 4DCT_GT_, but 4DCT_16_ is available clinically.

## 2 MATERIALS AND METHODS

### 2.1 Images and contours

**FIGURE 1:**
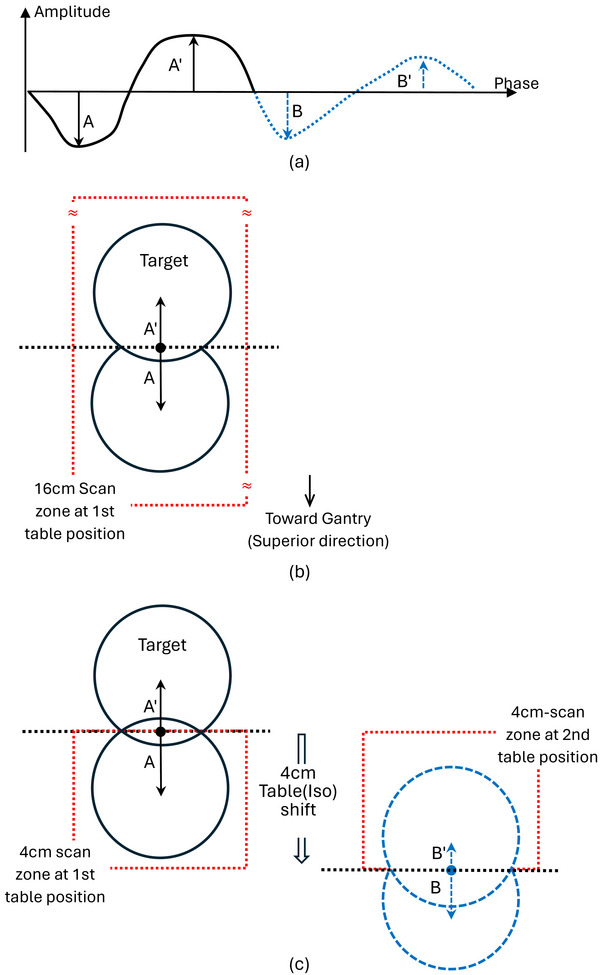
Phantom setup and motion driven by a respiratory trace for 4DCT acquisition performed in this study. (a). A respiration trace exhibiting a baseline shift and an amplitude reduction (A–B; A'–B') in its second period (blue‐dotted). (b). 4DCT_16_: Phantom motion in the superior–inferior direction, driven by the trace in its first period in (a), that is covered by the 16‐cm scan length. (c). 4DCT_4_: Phantom motion, driven by the trace in its first period in (a), which covers the superior part of the target, and by the trace in its second period, which covers the inferior part of the target (blue trace and target). For 4DCT_16_, within a single table position the full trend of motion of the first period only was captured: the amplitude of “A” is the maximum superior excursion of the target; “A'” is the maximum inferior excursion. For 4DCT_4_, in the first table position, the 4‐cm long scan captured the superior part only, while the target motion was driven by the first period, which is identical to the motion used for 4DCT_16_; after shifting the table superiorly by 4 cm, in the second position, the scan covered the inferior part, while the motion was driven by the second period with the reduced amplitudes of “B” and “B'”.

A lung‐mimicking phantom that contains a 3‐cm‐diameter cavity was placed with an equivalent‐sized sphere, and was used with ten respiratory traces taken from CyberKnife treatments. In addition, three morphologically tumor‐shaped targets were placed in the cavity and were used with their respective traces. Figure 1 depicts a respiratory trace (A) exhibiting an amplitude drop (A–B; A'–B') and a baseline shift (A–B). It is used to drive 1D target motion in the phantom during 4DCT acquisition. The magnitudes of the amplitudes were mapped directly to the physical motion range; specifically, the maximum excursion was defined by the maximum amplitudes, comparing A and A' between Figure 1a and 1b for 4DCT_16_. Following the respiratory trend, the target moved superiorly until the trace reached its valley (A) and inferiorly until it reached its peak (A'). Throughout the motion, the target placed in a single table position was encompassed by the 16 cm length, which is indicated by the dotted box. Unlike 4DCT_16_, 4DCT_4_ utilized two table positions, separated by a 4‐cm table shift, as shown in Figure 1c. During the first table position of the scan, the target moved following the first period‐portion of the trace; during the second position, after the shift, the target moved following the second period‐portion. Note that as the table shifted, the isocenter of the target (horizontal dotted line) shifted relative to the scanner. The 4‐cm long scan region, the red‐dotted box, captured the target superiorly when it was moving following the first‐period portion, but inferiorly when it was following the second‐period portion. When the two reconstructed images from the two scans were stitched together, differences between the first and second portions produced a discontinuity at the junction, the image artifacts observed in this study.

A total of thirteen cases were imaged when, using the selected traces, the targets were moved to each of the ten positions/phases in three different ways:

1. step‐wisely by helical scan at each movement (4DCT_GT_),
2. continuously by 4DCT_16_ axial scan, and
3. the same with 4DCT_4_ axial scan.

For #1 scan, mA was set using SmartmA 50–740, the pitch of 0.984:1, and a standard reconstruction kernel. For #2 and 3 scans, 250 mA and a standard reconstruction kernel. The choice of scan #1 as the GT (4DCT_GT_) was explained in the introduction. The trace of the first period was used for the GT scan as well as 4DCT_16_, while the trace of both periods was used for 4DCT_4_. Irregularities (baseline and/or amplitude change of the traces) affected #3 only after repositioning. The 4DCTs were phase‐sorted with a 15% tolerance. The motion extents derived from ten patient traces were 2.06, 1.18, 1.14, 0.83, 1.36, 2.13^^^, 1.58, 1.92^^^, 1.24^^^, and 1.87 in centimeters (^^^ also used with the tumor targets). The experimental steps and tumor shapes were described in our preceding work to this study.^8^ The iGTVs were contoured in maximum‐intensity‐projection (Mip) images of 4DCT_16_ as well as 4DCT_4_, and copied onto average‐intensity‐projection (Ave) images for each case.

### 2.2 Treatment planning

Treatment plans were generated, which included intensity‐modulated proton therapy (IMPT) and volumetric modulated arc therapy (VMAT) on Ave CT images of 4DCT_16_ and 4DCT_4_, respectively. The IMPT plans were targeted at left‐sided iGTVs with density overrides to 1.0 g/cm^3^, utilizing two beams from 45° and 130° with a 4‐cm range shifter for both beams. They were generated within the RayStation (RS) treatment planning system (RaySearch Laboratories, Sweden), employing Pencil Beam Scanning and the Monte Carlo Version 5.2 dose algorithm with the IBA Proteus Plus proton machine. A relative biological effectiveness of 1.1 was employed throughout. Robust planning with setup and density uncertainties was not used because the robustness of 4D doses with the uncertainties could not be evaluated in the RS system. Due to this limitation, the 4D dose coverage could be validated without robust evaluation, while planning was done similarly without robust optimization.

The VMAT plans were targeted at iGTVs+3 mm, forming planning target volumes (PTVs), employing two arcs from 340° to 180° and from 180° to 340° with collimation set to 90° for the latter arc only. An energy of 6 MV was used with a collapsed‐cone algorithm within the RS system. The plan objectives for both IMPT and VMAT were as follows: 60 Gy to the 95% volume of targets (*V*_prescribed dose(PD) _= 95%); 110% (66 Gy) reaching less than 1% of the target volumes, while minimizing the normal lung volume that received 20 Gy or less. The 110% maximum was chosen due to the complex shape of the tumor‐shaped targets.

### 2.3 4D dose calculation

The thirteen cases were imaged by the three scans (#1–3). The latter two images (#2 and 3) were planned by IMPT as well as VMAT, while #1 scan served as GT. Each plan, based on 4DCT_16_ and 4DCT_4_, was recalculated on individual phase images (GTV) of GT scans. The calculated phase‐specific doses were transferred to the maximum‐exhale image of each case, to which rigid registration of other phase images had been performed. Each registration was visually verified by assessing in terms of the geometry of the spherical target and that of the spherical cavity that contained the tumor targets. These doses were then summed, generating the 4D deliverable dose. Using the most accurate phase images of 4DCT_GT_ in representing moving target volumes (GTVs), the dose was obtained, and analyzed. Note that the other 4DCT images might have geometrical misses or artifacts, but plans based on them delivered 4D doses that can be calculated in the images of 4DCT_GT_. Therefore, the accuracies of the planning images of 4DCT_16_ and 4DCT_4_ in deliverable doses were respectively validated in terms of V_PD_ of the 4D dose in GTV for IMPT and average dose deposited in it. This effectively tested whether the coverage of iGTV encompassed that of GTV. A similar validation was done in PTV of phase 50 (maximum exhale) for VMAT. The dosimetry accuracy of 4DCT_4_ was further compared with that of 4DCT_16_ in terms of their 4D doses in 4DCT_GT_ in the dose coverages of targets, conformity indices (CIs), and homogeneity indices (HIs). The 4D dose based on 4DCT_16_ was also acquired using its own phase images, and was compared with that based on phase images of 4DCT_GT_, validating the dosimetry accuracy of the images of 4DCT_16_. The CIs were obtained, as defined in the RS system as

(1) $$\frac{\mathrm{target}\;\mathrm{volume}\;\mathrm{covered}\;\mathrm{by}\;\mathrm{the}\;95\%\;\mathrm{isodose}\;\mathrm{line}}{95\%\;\mathrm{isodose}\;\mathrm{volume}}.$$

The HIs were also obtained as

(2) $$\frac{\mathrm{dose}\;\mathrm{at}\;\mathrm{the}\;95\%\;\mathrm{target}\;\mathrm{volume}}{\mathrm{dose}\;\mathrm{at}\;5\%\;\mathrm{target}\;\mathrm{volume}}$$

where the 5% target volume is located at high‐dose tails. The dose and volume data were statistically tested by a paired *t*‐test, from which the corresponding *p*‐values were obtained.

## 3 RESULTS AND DISCUSSIONS

### 3.1 Characteristics of calculated doses

**FIGURE 2:**
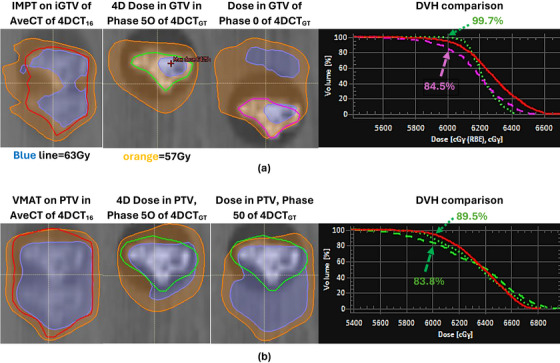
Dose distributions of IMPT and VMAT plans on various images of patient #6. The distributions included planned doses on Ave CT of 4DCT_16_, their 4D doses on phase 50 CT of 4DCT_GT_, and phase doses on phase 0 or 50 CT of 4DCT_GT_. The case of this patient was selected for illustrative purposes, whereas the distributions from all cases are evaluated in Tables 1, 2, 3 and Figure 3. iGTV and PTV are shown in red; GTV and its PTV in phase 50 CT in green; GTV in phase 0 CT in purple. For IMPT, *V*_PD_ increased from 84.5% (purple) in GTV at phase 0% to 99.7% (green) in GTV of the 4D dose, as shown in the DVH plot, Figure 2a. For VMAT, *V*_PD_ increased from 83.8% (dashed green) in PTV at phase 50% to 89.5% (dotted green) in PTV of the 4D dose, as shown in the DVH plot, Figure 2b. These data correspond to patient #6 in Table 1 and Figure 3. The phase 0 dose for IMPT and the phase 50 dose for VMAT were chosen to demonstrate undercoverages found in phase images.

This section compares the 4D doses with the 3D doses and the phase doses, shown in Figure 2, and characterizes the former to evaluate the 4DCTs.

Conformity: The conformity was shown to be worse with the 4D doses than with the 3D doses obviously because the latter, planned on iGTV for IMPT and its PTV for VMAT, were recalculated on the smaller targets of GTV and its PTV for IMPT and VMAT, respectively. Note that the latter PTV was created from GTV, thereby smaller than the former PTV. This was evidently visible from the relative size of the 57 Gy (95%) isodose lines to the sizes of the targets they covered, as shown in Figure 2a and b for IMPT and VMAT, respectively. The figure was provided for patient #6 with the tumor target when 4DCT_16_ was used for planning. The case of patient #6 was selected because it is one of the three tumor‐target cases that exhibited the largest under‐coverage in phase doses for both IMPT and VMAT, as stated in the legend of Table 1. It best illustrated the trend of 4D doses relative to phase doses, which was further evaluated later in this paper. Table 1 lists the calculated 4D doses for 4DCT_16_‐based planning with their evaluation indices in minimum, maximum, and average values from all spherical target cases and those from the three tumor target cases (patients #6, 8, and 9; the IDs were listed to relate them to our preceding study^8^). Table 2 lists them for 4DCT_4_‐based planning. The trend of the dose conformity was similar, although an isodose plot was not provided.

**TABLE 1:** Dose values of 4D recalculations of IMPT and VMAT plans, based on 4DCT_16_, performed in this study. IMPT plans were created on iGTVs of 4DCT_16_, but recalculated on phase‐specific GTVs of 4DCT_GT_ and those of 4DCT_16_, and summed, respectively, for each patient. Similarly, VMAT plans were created on PTVs (of iGTVs), but recalculated on PTVs (of GTVs), and summed. *D_a _* = average 4D dose in target; CI = Conformity index; HI = Homogeneity index. *V*_PD_ (%), *D_a_* (Gy), CI, and HI were averaged over ten sphere target cases with 10 respective respiratory traces (Ave_sphere_) and three tumor targets with their respective traces (Ave_tumor_). In addition, individual minimum and maximum values of each parameter were provided. P‐values for IMPT and VMAT were provided for Ave_sphere_ of each parameter, comparing 4DCT_16_ with 4DCT_GT_. The red‐colored data implies undercoverage in *V*_PD_ (< 95%). The blue‐colored fonts imply *p*‐values under 0.05 (i.e., statistically significant). The individual data of *V*_PD_ are shown in Figure 3. For IMPT plans on the spherical targets of patients #3 and 6–10, *V*_PD_ in the GTV of phase 0 fell below 95% with the minimum of 88.5% observed in patient #9; on two tumor targets (patients #6 and 8), *V*_PD_ was below 95% reaching the smaller value of 84.5% in patient #6. For VMAT plans on the spherical targets of patient #5–9, *V*_PD_ in the PTV of phase 0 or 50 was below 95% with the minimum of 86.5% in patient #6; on all tumor targets (patients #6, 8, and 9), *V*_PD_ was under 95% with the minimum of 80.5% in patient #6. The patient #s were identified here to refer to our preceding study.^8^ ^.^

	IMPT on 4DCT_16_								VMAT on 4DCT_16_							
	GTV of P50(4D)								PTV of P50(4D)							
	4DCT_16_				4DCT_GT_				4DCT_16_				4DCT_GT_			
Evaluation Indices	*V*_PD_	*D_a_*	CI	HI	*V*_PD_	*D_a_*	CI	HI	*V*_PD_	*D_a_*	CI	HI	*V*_PD_	*D_a_*	CI	HI
Min	98.38	62.39	0.63	0.94	96.00	62.34	0.53	0.94	93.50	63.14	0.71	0.91	93.50	63.27	0.65	0.91
Max	99.75	62.95	0.72	0.95	99.66	62.94	0.72	0.96	98.50	64.14	0.84	0.93	98.74	64.28	0.84	0.94
**Ave_sphere_**	98.92	62.76	0.67	0.95	98.95	62.76	0.64	0.95	96.60	63.79	0.80	0.92	97.26	63.90	0.76	0.93
**σ_sphere_**	1.10	0.17	0.03	0.01	1.09	0.19	0.05	0.01	1.71	0.29	0.04	0.01	1.60	0.28	0.06	0.01
P‐value	0.533	0.862	0.01	0.08					0.02	0.00	0.01	0.04				
Min	96.50	62.10	0.27	0.95	95.79	62.05	0.24	0.95	92.00	63.70	0.26	0.88	89.50	63.54	0.26	0.88
Max	99.67	62.47	0.51	0.96	99.73	62.53	0.52	0.96	98.50	64.77	0.66	0.90	99.24	64.86	0.66	0.91
**Ave_tumor_**	98.56	62.28	0.37	0.96	98.27	62.31	0.37	0.96	94.83	64.33	0.47	0.89	94.41	64.33	0.48	0.89

**TABLE 2:** Dose values of 4D recalculations of all plans, based on 4DCT_4_, performed in this study. The data in this table were similarly acquired to those in Table 1, except that 4D recalculation on 4DCT_4_ was not done. IMPT plans were created on iGTVs of 4DCT_4_, but recalculated on phase‐specific GTVs of 4DCT_GT_, and summed for each patient. VMAT plans were similarly created on PTVs (of iGTVs), but recalculated on PTVs (of GTVs), and summed. P‐values for IMPT were provided for Ave_sphere_ of each dose parameter, comparing 4DCT_GT_ calculations of 4DCT_4_‐based plans with 4DCT_GT_ calculations of 4DCT_16_‐based plans. P‐values for VMAT were similarly provided. The individual data of *V*_PD_ are shown in Figure 3. For IMPT plans on the spherical targets of patients #3, 5–6, and 8–10, *V*_PD_ in the GTV of phase 0 or 90 fell below 95% with the minimum of 45.8% in patient #10; on all tumor targets, *V*_PD_ was below 95% with the lowest of 57.0% in patient #8. For VMAT plans on the spherical targets of patient #3, 5–10, *V*_PD_ in the PTV of phase 0, 90, or 50 was below 95% with the minimum of 63.5% in patient #10; on all tumor targets, *V*_PD_ was below 95% with the minimum of 64.0% in patient #8.

	IMPT on 4DCT_4_				VMAT on 4DCT_4_			
	GTV of P50(4D)				PTV of P50(4D)			
	4DCT_GT_				4DCT_GT_			
Evaluation Indices	*V*_PD_	*D_a_*	CI	HI	*V*_PD_	*D_a_*	CI	HI
Min	70.42	60.69	0.52	0.83	79.50	62.46	0.60	0.83
Max	99.20	63.07	0.77	0.95	98.00	64.12	0.87	0.93
**Ave_sphere_**	93.83	62.44	0.68	0.93	93.20	63.54	0.76	0.91
**σ_sphere_**	9.29	0.68	0.09	0.04	5.81	0.48	0.10	0.03
P‐value	0.09	0.18	0.10	0.06	0.04	0.04	0.95	0.05
Min	96.00	61.36	0.38	0.95	87.50	63.58	0.52	0.87
Max	98.63	62.56	0.65	0.96	96.51	63.83	0.76	0.90
**Ave_tumor_**	97.38	61.94	0.48	0.95	91.17	63.73	0.65	0.88

**TABLE 3:** Gamma pass rates comparing 4D dose in 4DCT_16_ with that in 4DCT_GT_ of IMPT and VMAT plans generated on 4DCT_16_. In addition to the volumetric evaluation provided in Table 1, three‐dimensional Gamma factors were calculated for the three criteria of distance‐to‐agreement (mm) and dose difference (%). A 10% dose threshold was used.

	IMPT on 4DCT_16_			VMAT on 4DCT_16_		
	GTV of P50(4D)			PTV of P50(4D)		
Evaluation Indices	3D Gamma Pass Rates			3D Gamma Pass Rates		
	3 mm/3%	2 mm/2%	1 mm/1%	3 mm/3%	2 mm/2%	1 mm/1%
Min	98.42	94.39	89.09	98.06	92.25	82.32
Max	99.99	99.99	99.64	100.00	100.00	99.94
**Ave_sphere_**	99.83	99.28	97.46	99.80	99.15	97.45
**σ_sphere_**	0.50	1.72	3.09	0.61	2.43	5.43
Min	99.99	99.91	99.22	100.00	99.98	98.92
Max	100.00	99.97	99.86	100.00	100.00	99.99
**Ave_tumor_**	100.00	99.95	99.49	100.00	99.99	99.52

Homogeneity: The 4D dose was associated with a steeper gradient, compared with the 3D dose, and therefore greater homogeneity in the trend of its dose‐volume‐histogram (DVH), as presented in Figure 2a for the case of IMPT (HI was 0.96 for the 4D and 0.92 for the 3D). This trend was not observed when the 4D dose under‐covered the target with 89.50% in *V*_PD_, as shown in Figure 2b for VMAT (HI was 0.88 for the 4D and 0.90 for the 3D). Due to the sample size of the tumor targets, *σ* was not calculated. The trend of HI was not clearly found in the plans of 4DCT_4_ as well, due to undercoverages of targets by 4D doses, as shown by *V*_PD_s of Ave_sphere_ of 4DCT_GT_ for IMPT (93.83%) and VMAT (93.2%) and that of Ave_tumor_ for VMAT (91.17%) in Table 2. The undercoverages were further explained in the later section.

Volume coverage and dose maximum: When IMPT was used, *V*_PD_ was increased from 84.50% in GTV at phase 0 to 99.73% in GTV of the 4D dose, as presented in Figure 2a (see purple arrow vs. green arrow in the DVH plot). For VMAT, *V*_PD_ increased from 83.78% in PTV at phase 50% to 89.50% in PTV of the 4D dose, as shown in Figure 2b (see green dashed arrow vs. green dotted arrow in the DVH plot). Also, the dose maximum was decreased from 64.8 Gy in GTV at phase 0 to 64.0 Gy in GTV of the 4D dose, as shown in the high‐dose tail of DVH trends of Figure 2a for IMPT. A similar trend was observed for VMAT, as illustrated in Figure 2b. These changes in the volume coverage and the maximum dose can be explained as follows. The planned iGTV received a spatially inhomogeneous dose, as demonstrated by the slope of 3D dose DVH of IMPT in Figure 2a, while its GTV was moving. This motion deposited averaged, and therefore, a less inhomogeneous dose in GTV by reducing local hot spots and cold spots that occurred near its motion limits at phases 0 and 50. A similar explanation applies to PTVs in VMAT. The changes in the volume coverage and the maximum dose between the 3D doses in phase images and the corresponding 4D doses improved homogeneity for both IMPT and VMAT. However, between the 3D dose in iGTV and the 4D dose, this was found for IMPT only (when the undercoverage was not found) and not for VMAT.

### 3.2 4D doses: Under‐ or overdoses

#### 3.2.1 Plans based on 4DCT_16_

**FIGURE 3:**
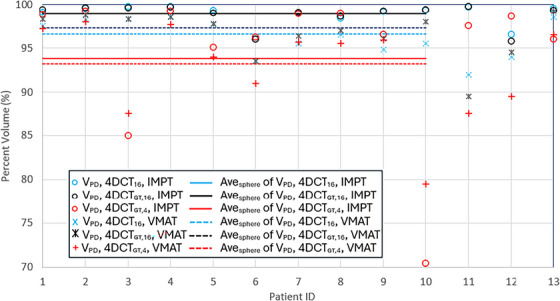
*V*_PD_ values of 4D calculations of IMPT and VMAT plans, based on 4DCT_16_ and 4DCT_4_, performed in this study. *V*_PD_, 4DCT_16_, IMPT denotes *V*_PD_ of the 4D dose calculated in 4DCT_16_ of IMPT plans generated on 4DCT_16_. Ave_sphere_ of *V*_PD_, 4D_16_, IMPT represents the averaged *V*_PD_ over the ten spherical‐target cases. *V*_PD_, 4DCT_GT,16_, IMPT denotes *V*_PD_ of the 4D dose calculated in 4DCT_GT_ of IMPT plans on 4DCT_16_. All other terms follow the same naming convention. The patient IDs of 11–13 (#6, 8, and 9, respectively) are the tumor‐target cases, which were not averaged due to the limited sample size. The standard‐deviation values for the average values are listed in Tables 1 and 2, and not depicted here to avoid a complex display.

Dose coverage of IMPT: The IMPT plans achieved an average GTV volume coverage of 98.95% with 95.69% at ‐3σ limit (95% confidence interval per Gaussian) for the spherical targets, as indicated by *V*_PD_ of Ave_sphere_ under 4DCT_GT_, GTV of P50 (4D) in Table 1 and shown in Figure 3 (see Ave_sphere_ of *V*_PD_, 4DCT_GT,16_, IMPT). Figure 3 displays *V*_PD_ values of all 4D calculations performed in this study, along with the average value of each case; the spherical target cases were identified with IDs of 1 through 10 and the tumor target cases were with 11–13. The tumor targets had an average coverage of 98.27%, similarly shown in Table 1. No individual cases exhibited *V*_PD_ values below 95%, as shown in Figure 3 (see the dark‐circle marker for *V*_PD_, 4DCT_GT,16_, IMPT); however, phase‐specific GTVs showed undercoverage in certain phases for multiple patients (patients #3 and 6–9 for the spherical targets; patients #6 and 8 for the tumor targets), as stated in the legend of Table 1 without each coverage value. This undercoverage was attributed to the smaller extension of 4DCT_16_ in the inferior direction for phase 0, compared with that of GT. The discrepancy was caused by sorting differences (experimental errors included) between the acquisitions of 4DCT_16_ and 4DCT_GT_, which associated different amplitudes, even if small, to each phase between the two. Motion averaging inherent in 4DCT_16_, not 4DCT_GT_, contributed to the differences as well (see the preceding study^8^). Contouring uncertainties of targets (GTVs vs. iGTVs) and the conformity of the prescribed isodose line to iGTV, particularly at the target's motion extremities, also played a role. These arguments applied to similar undercoverages found in all other plans of this study.

Dose coverage of VMAT: The VMAT plans yielded an average volume of 97.26% with 92.45% at ‐3σ limit for the 4D doses of the spherical targets, as listed in Table 1 [see Ave_sphere_ of *V*_PD_, 4DCT_GT_, PTV of P50(4D)] and shown in Figure 3 (see Ave_sphere_ of *V*_PD_, 4DCT_GT,16_, VMAT). Only in one patient (#6) did the coverage fall below the prescribed 95% volume, to 93.5%, as shown in Figure 3 (see *V*_PD_, 4DCT_GT,16_, VMAT). For the three tumor targets, the average was 94.41%, below the 95% threshold. The individual values were depicted in Figure 3. The undercoverages (< 95% volume) in phase images were found for PTVs for spherical targets in multiple patients (#5–9) and for all tumor targets.

4D dose versus phase dose of IMPT and VMAT: For both VMAT X‐ray and IMPT Proton plans, the undercoverages of PTVs and GTVs in phase images did not lead to those of 4D doses in PTVs and GTVs, respectively. This was found for patients #5, 7–9 for VMAT and #3 and 6–9 for IMPT (the undercoverage values of *V*_PD_ are in part provided in the legend of Table 1). This is because the undercovered parts of GTVs, mostly in P0 and P50 images, received compensatory full or even greater doses than the prescribed (from local hot spots in their 3D dose distributions), as they moved to more central positions in the other phase images. While the proton plans maintained the GTV coverage in 4D doses, as prescribed for iGTV (*V*_PD _= 95%), despite phase‐specific undercoverages, the photon plans did not with statistical significance, as indicated by the above 92.45% at the ‐3σ limit.

IMPT versus VMAT: The disparity between the proton and photon plans can be explained as follows. Each phase image exhibits greater contrast between GTV and the surrounding structures (air in the cavity space or lung in phase CT image) than that between iGTV and the surroundings (both are made of a mixture of target, air, and lung in Ave CT). Compared with protons, photons have a greater buildup effect in the air/lung‐GTV interface. The proton plans utilized the density override in iGTVs that reduces the contrast difference between Ave CT and phase CT images. These factors all combined influenced dose buildup differently between the IMPT and VMAT plans. Consequently, the proton plans met the prescription in the GTV coverage in 4D doses for all respective patients, but the photon plans did not, as shown in Figure 3. Considering that the dose calculation of VMAT was based on the collapsed‐cone model, if a dose‐calculation model reported to be more accurate in inhomogeneous media^22^ is used, the undercoverage in lung‐embedded targets can be more pronounced. Note that the undercoverages in certain phases found for the VMAT and IMPT plans based on 4DCT_16_, were also affected by contouring uncertainty and isodose conformity (stated previously).

#### 3.2.2 Plans based on 4DCT_4_

Dose coverage of IMPT and VMAT: The IMPT plans provided undercoverages with an average *V*_PD_ of 93.83% with 65.96% at ‐3σ limit for the spherical targets, provided under 4DCT_GT_, GTV of P50 (4D), and IMPT on 4DCT_4_ in Table 2 and shown in Figure 3 (see Ave_sphere_ of *V*_PD_, 4DCT_GT,4_, IMPT). Note the phase‐specific values of *V*_PD_, < 95%, for patients #3, 5, 6, and 8–10, that are in part disclosed in Table 2. The VMAT plans provided undercoverages with an average *V*_PD_ of 93.20% with 75.77% at ‐3σ limit for the spherical targets, due to the shorter extension (shorter motion ranges for patients #3, 5, and 10, provided in our preceding study^8^) and other reasons discussed above (difference between proton and photon). The coverages in *V*_PD_ were not only insufficient (< 95%), but also unreliable (large σ values) for IMPT and VMAT, as graphically found by the trend of the data for *V*_PD_, 4DCT_GT,4_, IMPT and *V*_PD_, 4DCT_GT,4_, VMAT in Figure 3. This implied inferiority to 4DCT_16_ when compared in *V*_PD_, *D_a_*, and HI of Ave_sphere_ under 4DCT_GT_, VMAT on 4DCT_16_ in Table 1, which is statistically supported by the significant *p*‐values (< 0.05). Unlike the case of VMAT, the support was not found for IMPT, due to the large variabilities of *V*_PD_s, *D_a_s*, CIs, HIs among patients (e.g., *σ*_sphere_ was 9.29% in *V*_PD_ in Table 2). The average volume coverage was 91.17% for the tumor targets [Ave_tumor_ under 4DCT_GT_, PTV of P50 (4D), VMAT on 4DCT_4_] that assumed geometrically irregular shapes, differently from the spherical targets. These results demonstrated the impact of the reported geometrical errors of 4DCT_4_^8–^ ^18^, ^23^ on dosimetry.

4D dose versus phase dose of IMPT and VMAT: It is noteworthy that despite significant undercoverages in GTVs at phases 0 and 50, the coverage of 4D dose was significantly greater (e.g., 70.42% vs. 45.8% for the spherical target, patient #10, with IMPT, Table 2 and Figure 3) due to the compensatory nature of the moving targets explained previously. Provided that the undercoverage occurred in phases 0 and 50 mostly, using a generous margin in the definition of iGTV and PTV may reduce the undercoverage, with a lesser extent for PTV (photons), particularly when 4DCT with short detector arrays is acquired. The average volume coverage was 97.38% for the tumor targets planned with IMPT (Ave_tumor_) with all individual targets exceeding the 95% coverage, despite undercoverages in certain phase‐specific GTVs, due to the compensatory 4D dose effects. However, this favorable trend was not as frequently observed as the IMPT plans for the VMAT plans with the average value of 91.17%, as noted above.

#### 3.2.3 Validation of 4DCT_16_ versus 4DCT_GT_

4DCT_16_ versus 4DCT_GT_: Between the two 4DCTs, no statistically significant difference was observed, as shown by the *p*‐values (> 0.05) of Ave_sphere_ of *V*_PD_, *D_a_*, and HI of the 4D doses based on 4DCT_16_, compared with those based on 4DCT_GT_, respectively, for IMPT. For instance, in Table 1, *V*_PD_, *D_a_*, and HI of 4DCT_16_, GTV of P50 (4D), and IMPT on 4DCT_16_ were 98.92%, 62.76 Gy, and 0.95, respectively, compared with 98.95%, 62.76 Gy, and 0.95 of 4DCT_GT_, GTV of P50 (4D), and IMPT on 4DCT_16_. This result showed that some errors in geometry and HU, reported by our prior study,^8^ did not make an impact on 4D dose between the two 4DCTs, and the level of agreement between the two^8^ was acceptable when it comes to 4D proton dose calculations. Although not statistically found for the case of VMAT, as shown by the *p*‐values of Ave_sphere_ of *V*_PD_, *D_a_*, and HI (< 0.05), their differences were small, < 1%, as shown by 96.60% versus 97.26%, 63.79 Gy versus 63.90 Gy, and 0.92 versus 0.93 in Table 1.

In addition to the volumetric evaluations, the dose differences were assessed spatially across target volumes three‐dimensionally (3D). 3D Gamma factors were calculated for various distance‐to‐agreement and dose difference criteria with 10% threshold (3 mm/3%, 2 mm/2%, and 1 mm/1%).^24^ With the exception of patient #10, excellent agreements were found with the minimum pass rates of 96.38% for the proton plan from patient #3 and 97.34% for the VMAT plan from patient #1 when the 1 mm/1% criterion was used; the lowest exceeded 99.5% across ten patients with the spherical targets when 2–3 mm/2–3% were used. This exception could be explained in part by the length of the target in his/her MIP image that was 4.9 mm greater than that of the ground truth, as reported in Table 1 (4D_16_‐GT) of our preceding study.^8^ This excessive length resulted in a sufficient target coverage in 4DCT_GT_ but with a lower CI compared with that in 4DCT_16_ (0.53 vs 0.64) as well as the pass rate of 89.09% when 1 mm/1% was used. As noted in Section 3.2.1, the excessive length was attributable to the sorting differences and the motion averaging inherent in 4DCT_16_; the contouring uncertainties of targets and the conformity of the prescribed isodose line additionally contributed to the reduced pass rate. All other patient cases did not exhibit the length differences greater than 2 mm, as shown in Table 1 of our preceding study, resulting in the excellent minimum pass rates reported above (96.38% and 97.34%). For the case of the tumor targets, the minimum pass rates were similarly high at 99.22% and 98.92% for IMPT and VMAT, respectively, when 1 mm/1% was used.

Impact of HU difference on dose: The HU difference was found to be 81.5 in the GTV image of the phase 80 of patient #6, which was greatest among all phases of all tumor targets, as shown in fig. 6 of our preceding work.^8^ The average VMAT dose was calculated to be 64.30 Gy in the PTV of the GTV in the phase image of 4DCT_16_, while it was 63.90 Gy in that of 4DCT_GT_, showing 0.6% difference. To understand this difference further, we overrode the HU of the GTV to that of water, which raised it by 412.3 HU, and recalculated. This resulted in 64.92 Gy showing a difference of 0.96%. The average IMPT dose was calculated to be 62.13 Gy in the GTV of the phase image of 4DCT_16_, while it was 62.05 Gy in that of 4D_GT_, showing 0.1% difference. The same override resulted in 62.51 Gy with a small difference of 0.61%. Based on the calculational models of the RS system, we could not find a meaningful difference in doses contributed by the HU difference found previously.^8^

## 4 FURTHER DISCUSSIONS

The motion ranges used in this study varied between 0.83 cm (for patient #4) and 2.13 cm (for patient #6), shown in Table 1 of our preceding study.^8^ Nine out of ten respiratory traces were associated with ranges greater than 1 cm. The ranges represented free‐breathing (FB) conditions used for CyberKnife tracking, and were comparable to the extents of lung tumor motion found in previous studies.^11^, ^12^ For example, Seppenwoolde et al.^11^ reported an average value of 1.2 ± 0.2 cm with a maximum of 2.46 cm in lung tumors; Yamamoto et al.^12^ reported 1.16 cm (range, 0.44–5.6 cm) in diaphragm or cardiac motion. The ranges therefore were appropriate for evaluating the accuracy of 4DCTs in this study. Various motion management techniques are available to reduce the motion range if the tracking is not employed. The range used therefore could exceed the usual range encountered in clinical practice when motion is compressed. In this regard when motion is compressed, the extent of the undercoverage in *V*_PD_ is likely to be smaller than those reported in this study. On the other hand, the agreement of 4DCT_16_ to 4DCT_GT_ would be even stronger than the already excellent agreement found in this study. When the respiration was more irregular than those of the ten traces chosen in this study, via baseline shifts and/or amplitude changes, a greater amount of underdoses than those reported in Section 3.2.2 could be found. The different build‐up effects between protons and photons were described in Section 3.2.1 for the comparative analysis of their 4D doses via *p*‐values. It is not clear how the build‐up difference affected the trend of *p*‐values.

When comparing 4DCT_16_ with 4DCT_GT_, the Gamma dose analysis, unlike the volumetric evaluation, revealed the discrepancy caused by the excessive target length of the former, as described in Section 3.2.3. This reflects the intrinsic limitation of the 4DCT (sorting uncertainty and others), as stated in Section 3.2.1, which remains applicable to 4DCT_16_. Unlike 4DCT_16_, we did not calculate 4D dose in 4DCT_4_. The decision was made because the image quality of individual phase images of 4DCT_4_ was substantially degraded by image misses, as demonstrated in Figure 2 of our preceding study.^8^ The calculation requires image registration between phase images and the maximum‐exhale image. The registration is essentially not feasible, and would be highly erroneous in the presence of these artifacts, regardless of the resulting dose outcome.

The 4DCT is an essential component in the management of moving targets during radiation therapy. The management may involve various steps of (1) 4D imaging, (2) treatment planning based on the acquired 4D images, (3) imaging prior to therapeutic radiation delivery, and (4) motion monitoring during the delivery.^25^, ^26^ The 4D imaging in step (1) is used for the conditions of free breathing (FB) and breathing under compression (CB). Steps (1) and (2) were fully utilized in the method section of this study. Through treatment imaging (3), after the patient is satisfactorily aligned anatomically, radiation delivery proceeds. If the alignment is inadequate, repeat imaging is performed, and the newly acquired images are evaluated in calculated planned doses via comparison with the original planned dose.^27^, ^28^ If this comparison indicates unacceptable deviation, replanning is initiated—an outcome more common in proton therapy than in photon therapy. The patient and target motion is monitored during radiation delivery (4), ensuring the extent of motion does not exceed that captured by the initial 4D imaging. Across all stages of management, the 4D images serve as the baseline reference for the subsequent steps, underscoring the importance of their accuracy. The 4DCT does not necessarily model the motion of a patient at the time of treatment, that may undergo changes during a fraction of treatment and/or between fractions of treatment, and therefore may be different from that at the time of the image acquisition. These changes were beyond the scope of this study.

The use of longer detector arrays in CT scanners enables extended longitudinal coverage and reduces imaging artifacts; however, it may also increase patient radiation exposure. Scanners with wider beam collimation can exhibit higher CTDIvol values in both axial and helical acquisition modes, primarily due to increased scatter radiation and the need to maintain uniform image quality across the expanded detector width. Although the final patient dose remains highly dependent on the specific imaging protocol, this potential pitfall warrants careful attention. Optimized acquisition parameters—such as appropriate tube current modulation, minimized scan length, and judicious selection of detector width—are essential to mitigate unnecessary dose and ensure patient safety.^29^

## 5 CONCLUSION

The 4DCT based on the longer detector array, 4DCT_16_, proved to be a reliable imaging method in providing accurate 4D dose by proton planning of IMPT when using Ave CT and Mip CT. However, it was less dependable for photon‐based VMAT planning, because of the required dose buildup at the interface between the targets (GTVs) and the neighboring materials (lung). In addition, the 4DCT based on 4DCT_16_ found to be indistinguishable from the ground‐truth 4DCT in 4D dose planning for IMPT, confirming its reliability. This finding was not equally applicable for VMAT with statistical significance via P‐values, but the former agreed with the latter by producing small differences (< 1%) (Section 3.2.3). However, the performance of 4DCT_16_ may be influenced by the intrinsic limitation of 4DCT imaging, such as phase‐sorting uncertainty.

In contrast, the 4DCT based on the shorter‐detector array, 4DCT_4_, was found to be unreliable in 4D deliverable dose by VMAT planning, due to its known geometrical inaccuracies and the inherent dose buildup requirements of photons (statistically proven). The use of 4DCT_4_ provided an inferior coverage to that of 4DCT_16_ for IMPT, not meeting the prescription in *V*_PD_, but the inferiority was not statistically supported.

Accurate 4D dose evaluation, needed for motion adaptive planning, is not possible when a limited‐length 4DCT is performed in the presence of respiration irregularity. However, this may be mitigated by generous delineation of targets. Alternatively, one can perform 4DCT when respiration becomes regular. The need of the 4DCT_16_ was shown by this study. However, the purchase of the 16 cm length CT needs consideration of benefit versus cost. Technically also, the purchase needs concurrent consideration of the entire motion management program of each institution, as described in our prior work.^8^ In institutions where the program is systematically structured to incorporate FB, CB, and breath‐holding, they can access the level of achievable patient alignment of each case prior to radiation delivery and that of the motion management via surface monitoring and/or tumor tracking during radiation delivery. Within such a framework, one can then clearly identify the necessity of 4DCT_16_ that functions as the foundational baseline for the entire motion management system. As the level of achievable accuracy in the system improves, the justification of 4DCT_16_ correspondingly increases.

## AUTHOR CONTRIBUTIONS

Inhwan Yeo has contributed to research planning, evaluation, and manuscript writing. Qianyi Xu has contributed to research planning, evaluation, and manuscript writing.

## CONFLICT OF INTEREST STATEMENT

The authors declare no conflicts of interest.

## References

[1] Rietzel E , Chen GTY , Choi N , Willet CG . Four‐dimensional image‐based treatment planning: target volume segmentation and dose calculation in the presence of respiratory motion. Int J Radiat Oncol Biol Phys. 2005;61(5):1535–1550. doi:10.1016/j.ijrobp.2004.11.037 15817360 10.1016/j.ijrobp.2004.11.037

[2] Meijers A , Knopf A‐C , Crijns APG . Evaluation of interplay and organ motion effects by means of 4D dose reconstruction and accumulation. Radiother Oncol. 2020;150:268–274. doi:10.1016/j.radonc.2020.07.055 32768509 10.1016/j.radonc.2020.07.055

[3] Yoon J , Jung JW , Kim JO , Yi B , Yeo I . Four‐dimensional dose reconstruction through in‐vivo phase matching of cine images of electronic portal imaging device. Med Phys. 2016;43(7):4420–4430. doi:10.1118/1.4954317 27370157 10.1118/1.4954317

[4] Yeo I , Jung JW , Yi B , Kim JO . Feasibility study on inverse four‐dimensional dose reconstruction using the continuous dose‐image of EPID. Med Phys. 2013;40(5):051702‐1‐11. doi:10.1118/1.4799941 10.1118/1.4799941 PMC4108721 23635250

[5] Admiraal MA , Schuring D , Hurkmans CW . Dose calculations accounting for breathing motion in stereotactic lung radiotherapy based on 4D‐CT and the internal target volume. Radiother Oncol. 2008;86(1):55–60. doi:10.1016/j.radonc.2007.11.022 18082905 10.1016/j.radonc.2007.11.022

[6] Flampouri S , Jiang SB , Sharp GC , Wolfgang J , Patel AA , Choi NC . Estimation of the delivered patient dose in lung IMRT treatment based on deformable registration of 4D‐CT data and Monte Carlo simulations. Phys Med Biol. 2006;51(11):2763–2779. doi:10.1088/0031‐9155/51/11/006 16723765 10.1088/0031-9155/51/11/006

[7] Rietzel E , Liu AK , Doppke KP , et al. Design of 4D treatment planning target volumes. Int J Radiat Oncol Biol Phys. 2006;66(1):287–295. doi:10.1016/j.ijrobp.2006.05.024 16904528 10.1016/j.ijrobp.2006.05.024

[8] Yeo I , Nie W , Fan J , Joo M , Correa M , Xu Q . Evaluating artifact‐free four‐dimensional computer tomography with 16 cm detector array. J Appl Clin Med Phys. 2025:e70056. doi:10.1002/acm2.70056 39989327 10.1002/acm2.70056 PMC11969080

[9] Barrett JF , Keat N . Artifacts in CT: recognition and avoidance. Radiographics. 2004;24(6):1679–1691. doi:10.1148/rg.246045065 15537976 10.1148/rg.246045065

[10] Coolens C , Webb S , Shirato H , Nishioka K , Evans PM . A margin model to account for respiration‐induced tumour motion and its variability. Phys Med Biol. 2008;53(16):4317–4330. doi:10.1088/0031‐9155/53/16/007 18653921 10.1088/0031-9155/53/16/007

[11] Seppenwoolde Y , Shirato H , Kitamura K , et al. Precise and real‐time measurement of 3D tumor motion in lung due to breathing and heartbeat, measured during radiotherapy. Int J Radiat Oncol Biol Phys. 2002;53(4):822–834. doi:10.1016/S0360‐3016(02)02803‐1 12095547 10.1016/s0360-3016(02)02803-1

[12] Yamamoto T , Langer U , Loo B , et al. Retrospective analysis of artifacts in four‐dimensional CT images of 50 abdominal and thoracic radiotherapy patients. Int J Radiat Oncol Biol Phys. 2008;72(4):1250–1258. doi:10.1016/j.ijrobp.2008.06.1937 18823717 10.1016/j.ijrobp.2008.06.1937 PMC2583232

[13] Han D , Bayouth J , Bhatia S , Sonka M , Wu X . Characterization and identification of spatial artifacts during 4D‐CT imaging. Med Phys. 2011;38(4):2074–2087. doi:10.1118/1.3553556 21626940 10.1118/1.3553556 PMC3078159

[14] Ge J , Santanam L , Noel C , Parikh PJ . Planning 4‐dimensional computed tomography (4DCT) cannot adequately represent daily intrafractional motion of abdominal tumors. Int J Radiat Oncol Biol Phys. 2013;85(4):999–1005. doi:10.1016/j.ijrobp.2012.09.014 23102840 10.1016/j.ijrobp.2012.09.014

[15] Watkins WT , Li R , Lewis J , et al. Patient‐specific motion artifacts in 4DCT. Med Phys. 2010;37(6Part1):2855–2861. doi:10.1118/1.3432615 20632597 10.1118/1.3432615

[16] Castillo SJ , Castillo R , Castillo E , et al. Evaluation of 4D CT acquisition methods designed to reduce artifacts. J Appl Clin Med Phys. 2015;16(2):23–32. doi:10.1120/jacmp.v16i2.4949 26103169 10.1120/jacmp.v16i2.4949 PMC4504190

[17] Mutaf YD , Antolak JA , Brinkmann DH . The impact of temporal inaccuracies on 4DCT image quality. Med Phys. 2007;34(5):1615–1622. doi:10.1118/1.2717404 17555243 10.1118/1.2717404

[18] Wink NM , Panknin C , Solberg TD . Phase versus amplitude sorting of 4D‐CT data. J Appl Clin Med Phys. 2006;7(1):77–85. 16518319 10.1120/jacmp.v7i1.2198 PMC5722473

[19] Coolens C , Bracken J , Driscoll B , Hope A , Jaffray D . Dynamic volume vs respiratory correlated 4DCT for motion assessment in radiation therapy simulation. Med Phys. 2012;39(5):2669–2681. doi:10.1118/1.4704498 22559637 10.1118/1.4704498

[20] Bissonnette JP , Balder PA , Dong L , et al. Quality assurance for image‐guided radiation therapy utilizing CT‐based technologies: A report of the AAPM TG‐179. Med Phys. 2012;39(4):1946–1963. doi:10.1118/1.3690466 22482616 10.1118/1.3690466

[21] Davis AT , Palmer AL , Nisbet A . Can CT scan protocols used for radiotherapy treatment planning be adjusted to optimize image quality and patient dose? A systematic review. Br J Radiol. 2017;90:20160406. doi:10.1259/bjr.20160406 28452568 10.1259/bjr.20160406 PMC5603945

[22] Webster M , Tanny S , Joyce N , et al. New dosimetric guidelines for linear Boltzmann transport equations through comparative evaluation of stereotactic body radiation therapy for lung treatment planning. J Appl Clin Med Phys. 2021;22(12):115–124. doi:10.1002/acm2.13464 10.1002/acm2.13464 PMC8664148 34783438

[23] Kwong Y , Mel AO , Wheeler G , Troupis JM . F our‐dimensional computed tomography (4DCT): A review of the current status and applications. J Med Imag Radiat Oncol. 2015;59(5):545–554. doi:10.1111/1754‐9485.12326 10.1111/1754-9485.12326 26041442

[24] Low DA , Harms WB , Mutic S , Purdy JA . A technique for the quantitative evaluation of dose distributions. Med Phys. 1998;25(5):656–661. doi:10.1118/1.598248 9608475 10.1118/1.598248

[25] Li H , Dong L , Bert C , et al. AAPM task group report 290: Respiratory motion management for particle therapy. Med Phys. 2022;49:e50–e81. doi:10.1002/mp.15470 35066871 10.1002/mp.15470 PMC9306777

[26] Purdie TG , Moseley DJ , Bissonnette J‐P , et al. Respiration correlated cone‐beam computed tomography and 4DCT for evaluating target motion in stereotactic lung radiation therapy. Acta Oncologica. 2006;45(7):915–922. doi:10.1080/02841860600907345 16982558 10.1080/02841860600907345

[27] Zhang Y , Jiang Z , Zhang Y , Ren L . A review on 4D cone‐beam CT (4D‐CBCT) in radiation therapy: Technical advances and clinical applications. Med Phys. 2024;51(8):5164–5180. doi:10.1002/mp.17269 38922912 10.1002/mp.17269 PMC11321939

[28] Qin A , Gersten D , Liang J , et al. A clinical 3D/4D CBCT‐based treatment dose monitoring system. J Appl Clin Med Phys. 2018;19(6):166–176. doi:10.1002/acm2.12474 30306710 10.1002/acm2.12474 PMC6236849

[29] Goldman LW . Principles of CT: Radiation dose and image quality. J Nucl Med Technol. 2007;35(4):213–225. doi:10.2967/jnmt.106.037846 18006597 10.2967/jnmt.106.037846

